# Extended spectrum β-lactamase and integron genes among *Shigella flexneri* and *Shigella sonnei* isolated from children with diarrhea in shiraz, Southwest Iran

**DOI:** 10.1186/s12887-025-06074-w

**Published:** 2025-10-21

**Authors:** Keyvan Farhadi, Abolfazl Rafati Zomorodi, Mohammadhassan Hassannezhad, Samane Mohebi, Mohammad Motamedifar, Leila Kasraian

**Affiliations:** 1 Education Development Center, Student Committee of Medical Education Development, Shiraz University of Medical Sciences, Shiraz, Iran; 2 Department of Bacteriology and Virology, School of Medicine, Shiraz University of Medical Sciences, Shiraz, Iran; 3 HIV/AIDS Research Center, Institute of Health, Shiraz University of Medical Sciences, Shiraz, Iran; 4 Blood Transfusion Research Center, High Institute for Research and Education in Transfusion Medicine, Tehran, Iran & Iranian Blood transfusion organization, Shiraz, Iran

**Keywords:** Antimicrobial resistance, ESBLs, Integrons, Multi-drug resistant, *Shigella* spp.

## Abstract

Shigellosis, a bacterial infection marked by dysentery, is typically a self-limiting disease and can be effectively managed with oral rehydration. However, antibiotics may help reduce the severity and duration of Shigellosis despite rising antimicrobial resistance. This study examines the prevalence of ESBL-producing isolates and the presence of *bla*_TEM_, *bla*_CTX−M_, *bla*_SHV_, *intI*1, and *intI*2 genes among *Shigella sonnei* and *Shigella flexneri* isolates from children with diarrhea in Shiraz, southwest Iran. From October 2019 to March 2020, 50 *S. flexneri* and 50 *S. sonnei were* isolated from children under 13 years old with diarrhea. Antimicrobial susceptibility and ESBL production were assessed, and the presence of ESBL-mediated genes, also *intI*1 and *intI*2 genes, was investigated using polymerase chain reaction methods. Resistance rates of 100% were observed against cefotaxime, ceftriaxone, streptomycin, and trimethoprim/sulfamethoxazole in all *S. flexneri* and *S. sonnei* isolates, with all isolates exhibiting multidrug resistance (MDR). ESBL positivity was found in (68%) of *S. flexneri* and (64%) of *S. sonnei* isolates. The *bla*_CTX−M_ gene was prevalent in (56%) of *S. flexneri* and (70%) of *S. sonnei* isolates. Remarkably, *intI*1 and *intI*2 were detected in (80%) and (86%) of *S. flexneri* isolates and (18%) and (90%) of *S. sonnei* isolates, respectively. In conclusion, the increasing resistance to first- and second-line antibiotics for treating shigellosis in Iran is a significant concern. The high prevalence of MDR *Shigella* spp. isolates in our region underscores the critical need to address the spread of antibiotic resistance and integrons in *Shigella* spp., making it an urgent priority.

## Introduction

Shigellosis is a bacterial infection characterized by dysentery, commonly referred to as dysentery. It is estimated that there are approximately 165 million cases of *Shigella*-related diarrhea annually, with 99% of these cases occurring in middle- and low-income countries, particularly affecting children (69%) [1]. Shigella genus comprises four species: *S. flexneri*, *S. sonnei*, *S. boydii*, and *S. dysenteriae*, encompassing over 50 serotypes. Among these, *S. flexneri* is the most prevalent species in developing countries, followed by *S. sonnei*, while *S. boydii* and *S. dysenteriae* are reported less frequently [2].

Shigellosis is primarily treated with supportive care and antimicrobial medications; however, there has been an increase in antimicrobial resistance (AMR) observed in *Shigella* strains [3]. A significant concern is the emergence of resistance to ciprofloxacin, the antibiotic the World Health Organization (WHO) recommended for shigellosis treatment. Additionally, a high level of resistance to other antibiotic classes—such as β-lactams, anti-metabolites, and tetracyclines—has been reported among *S. flexneri* and *S. sonnei* in several Asian countries [4, 5].

The production of extended-spectrum β-lactamase (ESBL) enzymes has emerged as a significant resistance mechanism among Gram-negative bacteria, including *Shigella* spp., conferring resistance to β-lactam antibiotics, particularly cephalosporins and monobactams [6, 7]. Generally, ESBL-producing bacteria often harbor genes that confer antibiotic resistance beyond β-lactams, leading to the development of multidrug-resistant (MDR) strains [8]. This phenomenon is associated with the presence of ESBL and other resistance genes on the same plasmids or other mobile genetic elements (MGEs), facilitating their transfer between bacterial species. Consequently, the increasing prevalence of ESBL-producing bacteria has resulted in higher morbidity rates, prolonged hospital stays, and more expensive treatment options [7, 9].

Integrons are recognized as genetic platforms capable of acquiring, integrating, and expressing gene cassettes that mediate AMR. Although integrons are not classified as MGEs, they are closely linked to MGEs, such as transposons, insertion sequences, and plasmids, significantly enhancing the transmission of these elements among bacteria [10].

The present study aims to investigate the prevalence of ESBL-producing strains and the presence of ESBL resistance genes, including *bla*_TEM_, *bla*_CTX−M_, and *bla*_SHV_, as well as integron classes 1 and 2 among *S. sonnei* and *S. flexneri* isolates from children with diarrhea in Shiraz, southwest Iran.

## Materials and methods

### Study design and participants

This cross-sectional study was conducted from October 2019 to March 2020 in Shiraz, Iran, one of the major cities in southwest Iran. During this time, 397 stool specimens were collected from patients with diarrhea referred to Shahid Dastgheib Educational Hospital of Shiraz, Iran. Shahid Dastgheib Educational and Therapeutic Center is affiliated to Shiraz University of Medical Sciences. Also, it is known as one of the leading centers for medical services provision to children in Shiraz, with 130 active beds and pediatric hospitalization, eyes, otorhinolaryngology, and children’s emergency wards. Stool specimens were obtained from patients under 13 years old with diarrhea; diarrhea stool specimens with blood and mucus in microscopic analysis were included in this study.

### Bacterial isolation and identification

Stool specimens were placed in Cary Blair transport medium and promptly transferred to the Microbiology Laboratory at Shiraz University of Medical Sciences for bacteriological analysis.

As described, specimens were cultured onto the Xylose lysine deoxycholate (XLD) agar medium and incubated at 37 ℃ for 24 h. Three to five pink colonies suspected of being Shigella spp. were assessed using Gram staining, catalase, and oxidase tests. The Gram-negative coccobacilli with catalase (+ ve) and oxidase (-ve) were subjected to further experiments, including Triple Sugar Iron (TSI) (to evaluate lactose fermentation and gas and glucose production), XLD (to assess lysine decarboxylation), Simon Citrate, methyl red/Voges-Proskauer(MR/VP), Sulfur-Indole-Motility (SIM) (to study the movement and production of Indole) and Urea (to study urea hydrolysis). Isolates that gave a positive methyl-red test with no lactose and gas production, no movement, negative urea hydrolysis, negative lysine decarboxylation, and negative citrate test were considered genus *Shigella*. To differentiate between Shigella species, a slide agglutination serotyping test was conducted using the *Shigella* polyvalent antisera obtained from Statens Serum Institute (MAST Group LTD, Merseyside, UK), following the manufacturer’s protocol.

### Antimicrobial susceptibility testing

The susceptibility of *S. flexneri* and *S. sonnei* isolates to 12 antibiotics was investigated using the Kirby-Bauer disc diffusion method recommended by CLSI guidelines. Such antibiotics were ceftazidime (30 µg), cefotaxime (30 µg), ceftriaxone (30 µg), tetracycline (30 µg), gentamicin (10 µg), amikacin (30 µg), chloramphenicol (30 µg), ofloxacin (5 µg), nalidixic acid (30 µg), ampicillin (10 µg), ciprofloxacin (5 µg) and streptomycin (10 µg) (HiMedia, India) [11]. *Escherichia coli* ATCC 25,922 and *Pseudomonas aeruginosa* ATCC 27,853 strains were used as quality control. Isolates resistant to at least one antibiotic of three antibiotic classes were considered as multi-drug resistance cases.

### Phenotypic detection of ESBL production

ESBL production was investigated using the Combined disc diffusion method (CDDM) according to the CLSI guideline [11]. In this regard, cefotaxime (30 µg) and ceftazidime (30 µg) alone and in combination with clavulanic acid (10 µg) (MAST Co., UK) were used. First, the isolate’s spread culture from a suspension (equivalent 0.5 MacFarland standard, 1-1.5 🞨 10^8^ CFU/mL) was prepared on the Muller Hinton agar (HiMedia, India). Then, discs were placed onto the plates within 20 mm distance and incubated at 35 ± 2 ℃ for 18–20 h. A positive result was interpreted if the inhibition zone around combined discs was ≥ 5 mm compared to the disc without clavulanic acid [7]. The negative and positive control strains were *E. coli* ATCC 25,922 and *Klebsiella pneumonia* ATCC 700,603, respectively.

### DNA extraction

The Boiling method was performed to extract DNA. Briefly, 300 µL of sterile distilled water was poured into each 1.5 mL Eppendorf vial, and 3–5 fresh colonies of incubated bacteria were dissolved. The vials were placed in a container at 100 °C for 10 min such that the boiling water covered two-thirds of the vials. The vials were then transferred to −20 °C for 10 min. Eventually, vials were centrifuged at 14,000 rpm for 5 min; 100 µL of supernatant was carefully transferred into the 500 µL Eppendorf vials as extracted DNA [12].

### Molecular detection of ESBL mediated genes and integron classes 1 and 2

All *S. flexneri* and *S. sonnei* isolates were subjected to the presence of three ESBL-mediated genes (including *bla*_TEM_, *bla*_CTX-M_, and *bla*_SHV_) and integron classes 1 and 2 using Uniplex-Polymerase chain reaction (PCR) technique. All PCR reaction was prepared in 25 µL final volume consisting of 12.5 µL of PCR 2× Master Mix (Amplicon, Denmark), 1 µL of each primer (2 µM as final concentration), 2 µL of template DNA, and up to a 25 µL final volume nuclease-free water. Table 1 presents the sequence of oligonucleotide primers, and the PCR conditions implied in this study.

**Table 1:** Oligonucleotide sequence used for determining ESBLs mediated genes and classes 1 and 2 integrons among *Shigella* spp.

Primers’ Name	Primer Sequence (5′→3′)	Size (bp)	Annealing (℃)	References
*bla*_TEM_	TCGCCGCATACACTATTCTCAGAATGA ACGCTCACCGGCTCCAGATTTAT	455	61	[43]
*bla*_CTX−M_	ATGTGCAGCACCAGTAAAGTGATG GC TGGGTAAAGTAAGTGACCAGAATC	593		
*bla*_SHV_	AGCGGATGCGTTATATTCGCCTG TG TGCTTTGTTATTCGGGCCAA	747		
*int*I1	CCTCCCGCACGATGATC TCCACGCATCGTCAGGC	280	55	[44]
*int*I2	TTATTGCTGGGATTAGGC ACGGCTACCCTCTGTTATC	233	50	

Abbreviation: *ESBLs* extended-spectrum β-lactamases

### Statistical analysis

The data were statistically analyzed using SPSS 25. Descriptive tests were used to investigate the frequency of each characteristic. The Chi-square test was undertaken to determine whether there was a significant relationship between groups; the *p-value* threshold for statistical significance was ≤ 0.05.

## Results

### Shigella isolation, antimicrobial susceptibility testing, and ESBLs detection

**Fig. 1:**
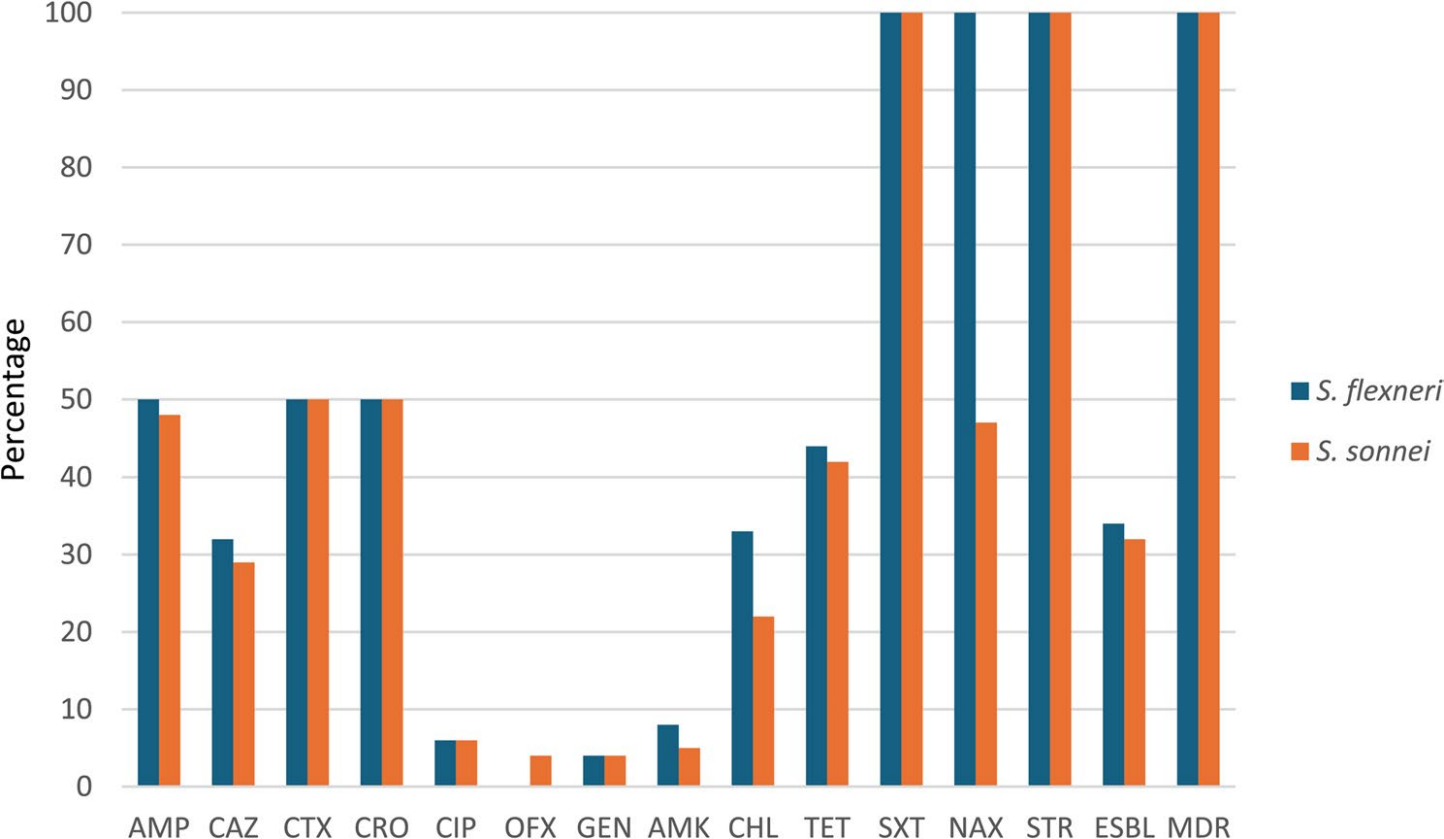
The prevalence of antimicrobial resistance among *S. flexneri* and *S. sonnei* isolates. Abbreviation: *CAZ* ceftazidime; *CTX* cefotaxime; *TET* tetracycline; *CRO* ceftriaxone; *GEN* gentamicin; *CHL* chloramphenicol; *OFX* ofloxacin; *NAX* nalidixic acid; *SXT* trimethoprim/sulfamethoxazole; *AMK* amikacin; *AMP* ampicillin; *CIP*; ciprofloxacin; *STR* streptomycin; *MDR* multi-drug resistant; *ESBL* extended-spectrum beta-lactamase

A total of 50 *S. flexneri* and 50 *S. sonnei* isolates were isolated from 397 diarrhea stool specimens. The highest resistance rates were determined to cefotaxime, ceftriaxone, streptomycin, and trimethoprim/sulfamethoxazole with 100% frequency among all *S. flexneri* and *S. sonnei* isolates. The prevalence of ESBL-producing isolates was 34/50 (68%) and 32/50 (64%) among *S. flexneri* and *S. sonnei*, respectively. Additionally, all *S. flexneri* and *S. sonnei* isolates were MDR (Fig. 1).

### Prevalence of ESBL mediated genes and integron classes 1 and 2

Generally, *bla*_CTX−M_ and *bla*_TEM_ were determined among 28/50 (56%) and 19/50 (38%) of the *S. flexneri* isolates, respectively; *bla*_SHV_ was detected in one *S. flexneri* isolate. The *intI*1 and *intI*2 genes were detected among 40/50 (80%) and 43/50 (86%) of *S. flexneri* isolates (Table 2).

**Table 2:** The prevalence of positive resistance genes in accordance with the antimicrobial resistance profile among *S. flexneri* isolates (*N* = 50)

Antibiotics		bla_CTX−M_ *N* = 28 (%)	*p* value^*^	bla_TEM_ *N* = 19 (%)	*p* value	bla_SHV_ *N* = 1 (%)	*p* value	intI1 *N* = 40 (%)	*p* value	intI2 *N* = 43 (%)	*p* value
CAZ	R	22 (78.6%) 0 6 (21.4%)	**0.01**	16 (84.2%) 0 3 (15.8%)	**0.03**	0 0 1 (100%)	-	22 (55%) 0 18 (45%)	0.39	25 (58.1%) 0 18 (41.9%)	0.96
	I										
	S										
CTX	R	28 (100%) 0 0	-^**^	19 (100%) 0 0	-	1 (100%) 0 0	-	39 (97.5%) 0 1 (2.5)	0.61	42 (97.7%) 0 1 (2.3%)	0.58
	I										
	S										
TET	R	24 (85.7%) 0 4 (14.3%)	0.57	16 (84.2%) 0 3 (15.8%)	0.52	1 (100%) 0 0	-	35 (87.5%) 0 5 (12.5%)	0.82	38 (88.4%) 0 5 (11.6%)	0.84
	I										
	S										
CRO	R	28 (100%) 0 0	-	19 (100%) 0 0	-	1 (100%) 0 0	-	40 (100%) 0 0	-	43 (100%) 0 0	-
	I										
	S										
GEN	R	4 (14.3%) 0 24 (85.7%)	0.06	2 (10.5%) 0 17 (90.5%)	0.61	0 0 1 (100%)	-	2 (5%) 0 38 (95%)	0.11	4 (9.3%) 0 39 (90.7%)	0.26
	I										
	S										
CHL	R	21 (75%) 0 7 (25%)	0.13	16 (84.2%) 0 3 (15.8%)	**0.03**	1 (100%) 0 0	-	25 (62.5%) 0 15 (37.5%)	0.29	29 (67.4%) 0 14 (32.6%)	0.59
	I										
	S										
OFX	R	0 0 28 (100%)	-	0 0 19 (100%)	-	0 0 1 (100%)	-	0 0 40 (100%)	-	0 0 43 (100%)	-
	I										
	S										
NAX	R	28 (100%) 0 0	-	19 (100%) 0 0	-	1 (100%) 0 0	-	40 (100%) 0 0	-	43 (100%) 0 0	-
	I										
	S										
SXT	R	28 (100%) 0 0	-	19 (100%) 0 0	-	1 (100%) 0 0	-	40 (100%) 0 0	-	43 (100%) 0 0	-
	I										
	S										
AMK	R	5 (17.9%) 0 23 (82.1%)	0.68	1 (5.3%) 0 18 (94.7%)	0.08	0 0 1 (100%)	-	6 (15%) 0 34 (85%)	0.7	8 (18.6%) 0 35 (81.4%)	0.21
	I										
	S										
AMP	R	28 (100%) 0 0	-	19 (100%) 0 0	-	1 (100%) 0 0	-	40 (100%) 0 0	-	43 (100%) 0 0	-
	I										
	S										
CIP	R	3 (10.7%) 0 25 (89.3%)	0.75	1 (5.3%) 0 18 (94.7%)	0.22	0 0 1 (100%)	-	6 (15%) 0 34 (85%)	0.09	6 (14%) 0 37 (86%)	0.29
	I										
	S										
STR	R	28 (100%) 0 0	-	19 (100%) 0 0	-	1 (100%) 0 0	-	40 (100%) 0 0	-	43 (100%) 0 0	-
	I										
	S										
ESBL	+ve	28 (100%) 0	-	19 (100%) 0	-	0 1 (100%)	-	27 (67.5%) 13 (32.5%)	0.88	30 (69.8%) 13 (30.2%)	0.66
	-ve										

Abbreviation: *CAZ* ceftazidime; *CTX* cefotaxime; *TET* tetracycline; *CRO* ceftriaxone; *GEN* gentamicin; *CHL* chloramphenicol; *OFX* ofloxacin; *NAX* nalidixic acid; *SXT* trimethoprim/sulfamethoxazole; *AMK* amikacin; *AMP* ampicillin; *CIP* ciprofloxacin; *STR* streptomycin; *MDR* multi-drug resistant; *ESBL* extended-spectrum beta-lactamase; +*ve* positive; -*ve* negative

^*^p value ≤ 0.05 is significant

^**^ Not applicable

Also, 35/50 (70%), 31/50 (62%), and 2/50 (4%) of *S. sonnei* isolates containing the *bla*_CTX−M_, *bla*_TEM_, and *bla*_SHV_ genes, respectively. Surprisingly, *intI*1 and *intI*2 integrons were harbored by 45/50 (90%) and 9/50 (18%) of *S. sonnei* isolates, respectively (Table 3). 27/50 (54%) of *S. flexneri* isolates co-harbored the *bla*_CTX−M_ and *intI*2, indicating statistically significant co-occurrence (*p* = 0.017). Moreover, class 2 integron was present in 30/50 (60%) *S. sonnei* isolates containing the *bla*_TEM_ gene. A significant relationship was observed between class 2 integron and *bla*_TEM_ genes in the isolates studied (*p* = 0.041).

**Table 3:** The prevalence of positive resistance genes in accordance with the antimicrobial resistance profile among *S. sonnei* isolates (*N* = 50)

Antibiotics		bla_CTX−M_ *N* = 35 (%)	*p* value^*^	bla_TEM_ *N* = 31 (%)	*p* value	bla_SHV_ *N* = 1 (%)	*p* value	intI1 *N* = 9 (%)	*p* value	intI2 *N* = 45 (%)	*p* value
CAZ	R	24 (68.6%) 0 11 (31.4%)	0.3	20 (64.5%) 0 11 (35.5%)	0.92	1 (50%) 0 1 (50%)	-	6 (66.7%) 0 3 (33.3%)	0.85	29 (64.4%) 0 16 (35.6%)	0.84
	I										
	S										
CTX	R	35 (100%) 0 0	-	31 (100%) 0 0	-	2 (100%) 0 0	-	9 (100%) 0 0	-	45 (100%) 0 0	-
	I										
	S										
TET	R	29 (82.9%) 0 6 (17.1%)	0.73	26 (83.9%) 0 5 (16.1%)	0.97	2 (100%) 0 0	-	7 (77.8%) 0 2 (22.2%)	0.58	37 (82.2%) 0 8 (17.8%)	0.17
	I										
	S										
CRO	R	35 (100%) 0 0	-	31 (100%) 0 0	-	2 (100%) 0 0	-	9 (100%) 0 0	-	45 (100%) 0 0	-
	I										
	S										
GEN	R	2 (5.7%) 0 33 (94.3%)	0.38	3 (9.7%) 0 28 (90.3%)	0.56	0 0 2 (100%)	-	1 (11.1%) 0 8 (89.9%)	0.71	4 (8.9%) 0 41 (91.1%)	0.34
	I										
	S										
CHL	R	17 (48.6%) 0 18 (51.4%)	0.32	13 (41.9%) 0 18 (58.1%)	0.7	2 (100%) 0 0	-	2 (22.2%) 0 7 (77.8%)	0.14	20 (44.4%) 0 25 (55.6%)	0.84
	I										
	S										
OFX	R	4 (11.4%) 0 31 (88.6%)	0.17	4 (12.9%) 0 27 (86.1%)	**0.04**	0 0 2 (100%)	-	0 0 9 (100%)	-	4 (8.9%) 0 41 (90.9%)	0.34
	I										
	S										
NAX	R	34 (97.1%) 0 1 (2.9%)	0.17	29 (93.5%) 0 2 (6.5%)	0.86	2 (100%) 0 0	-	9 (100%) 0 0	-	43 (95.6%) 0 2 (4.4%)	0.24
	I										
	S										
SXT	R	35 (100%) 0 0	-	31 (100%) 0 0	-	2 (100%) 0 0	-	9 (100%) 0 0	-	45 (100%) 0 0	-
	I										
	S										
AMK	R	4 (11.4%) 0 31 (88.6%)	0.6	3 (9.7%) 0 28 (90.3%)	0.92	0 0 2 (100%)	-	2 (22.2%) 0 7 (77.8%)	0.17	4 (8.9%) 0 41 (91.1%)	0.47
	I										
	S										
AMP	R	33 (94.3%) 0 2 (5.7%)	0.34	30 (96.8%) 0 1 (3.2%)	0.72	2 (100%) 0 0	-	9 (100%) 0 0	-	43 (95.6%) 0 2 (4.4%)	0.51
	I										
	S										
CIP	R	4 (11.4%) 0 31 (88.6%)	0.84	2 (6.5%) 0 29 (93.5%)	0.12	0 0 2 (100%)	-	0 0 9 (100%)	0.11	4 (8.9%) 0 41 (91.1%)	0.08
	I										
	S										
STR	R	35 (100%) 0 0	-	31 (100%) 0 0	-	2 (100%) 0 0	-	9 (100%) 0 0	-	45 (100%) 0 0	-
	I										
	S										
ESBL	+ve	29 (82.9%) 6 (17.1%)	**< 0.001**	24 (77.4%) 7 (22.6%)	**0.02**	2 (100%) 0	-	5 (55.6%) 4 (46.4%)	0.56	32 (71.1%) 13 (28.9%)	0.001
	-ve										

Abbreviation: *CAZ* ceftazidime; *CTX* cefotaxime; *TET* tetracycline; *CRO* ceftriaxone; *GEN* gentamicin; *CHL* chloramphenicol; *OFX* ofloxacin; *NAX* nalidixic acid; *SXT* trimethoprim/sulfamethoxazole; *AMK* amikacin; *AMP* ampicillin; *CIP* ciprofloxacin; *STR* streptomycin; *MDR* multi-drug resistant; *ESBL* extended-spectrum beta-lactamase; +*ve* positive; -*ve* negative

^*^p value ≤ 0.05 is significant

^**^ Not applicable

## Discussion

Diarrheal diseases are responsible for over one million deaths each year worldwide, with approximately 164,000 of these deaths attributed to shigellosis, particularly among children under the age of five. Furthermore, it is estimated that *Shigella* spp. cause more than 125 million episodes of diarrhea annually [3, 13]. Among those under five years of age, child diarrhea-related deaths account for 21%, and Africa and South Asia are still placed at the top of the list of child deaths [14].

Most diarrhea episodes are self-limiting and can be treated with oral rehydration therapy. However, medications can decrease the severity and length of certain etiologies, such as shigellosis caused by *Shigella* spp. Therefore, the WHO updated 2014 Integrated Management of Childhood Illness (IMCI) recommendations to treat suspected *Shigella* spp. infections with severe diarrhea with antibiotics [15, 16]. According to the WHO, *Shigella* isolated from Asia and Africa are becoming more resistant to fluoroquinolones and third-generation cephalosporins. Therefore, the WHO encourages antibiotic sensitivity research in these areas [17].

WHO lists ciprofloxacin as the first-line treatment choice for bloody diarrhea shigellosis. In the current study, the prevalence of resistance to ciprofloxacin was 24% and 12% among *S. flexneri* and *S. sonnei* isolates, respectively. Our finding was confirmed by previous investigations in Iran that reported ciprofloxacin resistance in the range of 14.7% − 17.8% and 2.7% − 31.8% among *S. flexneri* and *S. sonnei* isolates, respectively [18, 19]. However, these results were higher than earlier investigations in Iran that reported a frequency of resistance to ciprofloxacin at the rates of 1.4% and 0% among *S. flexneri* and *S. sonnei* isolates, respectively [20]; therefore increasing the prevalence of resistance to the first-line treatment choice, ciprofloxacin, is significant in Iran. This rise is in line with other surveys in Bangladesh [21], France [22], and Spain [23].

Remarkably, 100% of *S. flexneri* and *S. sonnei* isolates were identified as resistant to ceftriaxone, a second-line antibiotic treatment for shigellosis. Although previous studies have determined lower resistance to ceftriaxone among *S. flexneri* (52%, 41%) and *S. sonnei* (63.8%, 50%) isolates in Iran, there is still high frequency of resistance to ceftriaxone [19, 20]. A recent systematic review and meta-analysis study supported the increasing resistance to ciprofloxacin and ceftriaxone in Iran, which was recognized to grow from 0 to 6% and 6–42%, respectively, among *Shigella* spp. in Iran during this time [24]. However, further studies require investigating a larger sample size collected from across Iran and also evaluating susceptibility to azithromycin.

Continuously, the evaluation results of antibiotic sensitivity also revealed 100% resistance to cefotaxime, streptomycin, and trimethoprim/sulfamethoxazole among *S. flexneri* and *S. sonnei* isolates. The high frequency of *S. flexneri* and *S. sonnei* resistance to trimethoprim/sulfamethoxazole agreed with other studies in Iran [19, 20], as well, in Australia [25], India [26], USA [27], and Africa [28]. This comparison showed highlighted resistance to trimethoprim/sulfamethoxazole among *S. flexneri* and *S. sonnei* isolates worldwide.

In recent years, there has been a notable global rise in the prevalence of ESBL-producing Enterobacteriaceae, including *Shigella* spp.; patients infected with these ESBL-producing bacteria often experience unfavorable clinical outcomes, primarily due to delays in timely and effective antimicrobial treatment and the limited availability of therapeutic options [29]. The prevalence of ESBL positive was 68% and 64% for *S. flexneri* and *S. sonnei* isolates, respectively. These results have similarity with several prior publishments in Iran that indicated the prevalence of ESBL-producing *Shigella* spp. in the range of 43% − 54.2% [30–33], and most of them detected higher frequency of ESBL producer in *S. flexneri* isolates. In contrast, Sabour et al. found a lower frequency of *Shigella* spp. ESBL producer in northwest Iran, 10.2% [34]. Previous investigations in Asia have shown a lower incidence of ESBL producer *Shigella* spp., in India at 19% [35], in China at 25.8% [36], in Thailand at 20.9% [37], compared to what was published from Iran. Also, this comparison is supported by a systematic review and meta-analysis survey; it is determined an overall pooled frequency of ESBL-producing *Shigella* spp. in Asia 23% [4]. The observed differences in the frequency of ESBL-producing *Shigella* spp. may result from variations in detection methods, geographical locations, and differences in sample size, type, and study participants.

Evaluation of the presence of ESBL-mediated genes demonstrated the *bla*_CTX−M_ as the predominant one among *S. flexneri* (56%) and *S. sonnei* (70%) isolates, followed by *bla*_TEM_ with frequencies of 38% and 62%, respectively. These results were consistent with the previously found frequency of *bla*_CTX−M_ and *bla*_TEM_ by Ling-Zhang et al. in China, 91.8% and 3% [38]; Sabour et al. in Iran, 65.3% and 61.2% [34];; Sriyapai et al. in Thailand, 73.5% and 64.1% [37], respectively.

The genetic elements contributing to MDR phenotypes in *Shigella* spp. have been linked to the presence of various plasmids, integrons, and genomic islands. Integrons are especially noteworthy because of their capability to acquire, integrate, and spread resistance genes among bacteria, facilitating gene transfer both within and between species, which raises substantial concerns [39, 40]. This study detected the *intI*1 and *intI*2 among 80% and 86% of *S. flexneri* isolates and 18% and 90% of *S. sonnei* isolates, respectively. Also, all isolates in both *S. flexneri* and *S. sonnei* were MDR. Our findings indicate that the occurrence of class 2 integrons is significantly greater than that of class 1. This fact corroborates with earlier studies that reported a higher prevalence of class 2 integrons than class 1 among *Shigella spp.* isolates [41, 42].

A notable correlation was found between the prevalence of *intI*2 genes and MDR among the *Shigella* spp. isolates. These results imply a connection between the *intI*2 gene and other antibiotic resistance determinants, underscoring the need for more in-depth molecular investigations. Furthermore, ongoing surveillance studies in diverse global regions are essential to accurately assess the distribution of *Shigella* spp. isolates containing the *intI*2 gene.

Finally, several potential limitations need to be considered. First, more comprehensive investigations that include a larger sample size and a broader range of antibiotic families would provide further insights into the antimicrobial resistance (AMR) profiles among *Shigella spp.* isolates. Secondly, determining the molecular pathways of resistance gene dissemination, such as through plasmid replicon typing, could be beneficial for developing novel strategies to mitigate this process.

## Conclusion

Based on the findings of this study, we observed a high prevalence of AMR among *Shigella* isolates in Iran, with *S. flexneri* and *S. sonnei* demonstrating MDR in all cases. Alarmingly, resistance rates to cefotaxime, ceftriaxone, streptomycin, and trimethoprim/sulfamethoxazole were 100% among all isolates, underscoring the limited efficacy of these antibiotics in treating shigellosis in this region. While ofloxacin, gentamycin, and ciprofloxacin retained some effectiveness against *S. flexneri* and *S. sonnei*, the elevated resistance to ciprofloxacin, a primary treatment choice, is particularly concerning. In addition, a significant proportion of the isolates were ESBL producers. Routine monitoring programs are essential to track MDR *Shigella* strains and curb further dissemination, especially given the widespread MDR and high prevalence of ESBL-producing and integron-carrying isolates observed in this study.

## Data Availability

The datasets created and examined for this research can be obtained by reaching out to the corresponding author.

## Abbreviations

S. flexneri: Shigella flexneri
S. sonnei: Shigella sonnei
WHO: World Health Organization
ESBL: extended-spectrum β-lactamase
MDR: Multi-drug resistant
MGE: Mobile gene element
AMR: Antimicrobial resistance
CLSI: Clinical and Laboratory Standard Institute
PCR: Polymerase chain reaction
